# Familial Aggregation of Uterine Myomas in Japanese Women

**DOI:** 10.2188/jea.12.249

**Published:** 2007-11-30

**Authors:** Fumio Sato, Mitsuru Mori, Motoi Nishi, Ryuichi Kudo, Hirotsugu Miyake

**Affiliations:** 1 Department of Public Health, School of Medicine, Sapporo Medical University, Sapporo, Japan.; 2 Department of Obstetrics and Gynecology, School of Medicine, Sapporo Medical University, Sapporo, Japan.; 3 Department of Obstetrics and Gynecology, Junjindo Yuza Hospital, Yamagata, Japan.

**Keywords:** uterine myomas, familiality, nulliparity

## Abstract

To assess the familial aggregation of uterine myomas in Japanese women with myomas, one hundred forty four women requiring surgery for myomas and 288 age-matched healthy controls were studied in Hokkaido, Japan. The incidence of positive first-degree family history of myomas among women aged 45-54 years with myomas was greater than that among controls (31.5% versus 15.2%, respectively, *p* < 0.01). Analyses categorized by the status of parity and familiality among subjects showed that the risk for myomas was the greatest in women who had both fewer births (parity = 0 or 1) and the positive family history of myomas as compared with those who had both more births (parity ≧ 2) and the negative familiality of myomas (odds ratio = 5.8, 95% confidence interval = 2.3 – 14.6). The results of this study suggest that Japanese middle-aged women with myomas have the familial predisposition of uterine myomas. Furthermore, nulliparous women with the familial aggregation of myomas may be at increased risk of the disease.

## INTRODUCTION

In most countries, a major women’s health problem is present in the development of uterine leiomyomas. These benign common tumors have frequently been treated surgically among women in the reproductive years^1^^)^. Until recently, nevertheless the epidemiologic investigation of uterine myomas has not been fully studied^2^^,^^3^^)^. Clinical observations have suggested an association between uterine myomas and the family history of this disease. In addition, genetic studies have been carried out concerning the formation of uterine myomas^4^^,^^5^^)^. Few epidemiologic reports, however, have studied the familial predisposition of myomas^6^^,^^7^^)^. On the other hand, there is growing evidence that uterine myomas are associated with decreased reproductive outcomes, particularly nulliparity is a significant risk factor for uterine myomas^8^^,^^9^^)^. Although it seems to be important to estimate the impact of family history on parity for myomas, no relevalent studies have been reported. Therefore, to examine if Japanese women with uterine myomas have the familial aggregation of this disease and further, to evaluate the relationship between fertility and familiality we conducted an epidemiologic study in Hokkaido, Japan.

## METHODS

The present report was studied in Hokkaido, Japan between 1994 and 1995. The case group was one hundred fifty three women aged 30-54 years. All of them had uterine myomas with abnormal uterine bleeding, pelvic pain and/or pressure and had no other gynecological diseases. They required surgery because of the presence of these tumors and were confirmed pathologically of leiomyomas in Sapporo Medical University Hospital or its affiliated hospitals. Because of the refusal of participating in the study in 9 myoma patients, 144 women (94.1%) were enrolled. They underwent hysterectomy (91.0%) or myomectomy (9.0%) for this disease.

We recruited as controls women over 30 years of age, who paid a visit to the Detection Center of Hokkaido Cancer Society located in Sapporo and took part in a screening program for cervical cancer and breast cancer in the same period as that of the myoma patients. The elder controls, ≧ 55 years of age, were excluded from the present study because of no age-matched patients with myomas. If women had any past history of gynecological diseases, they were not recruited as control subjects. Thus, 354 healthy women aged 30-54 years participated in this study. Of these, 325 women (91.8%) consented to participate. Two control subjects were randomly selected for each case matched for age (± 2 years), consequently, 288 control subjects were eligible for this research. We asked a woman whether it had been pointed out by a doctor previously that she had myoma. Further, all of participants had the pelvic examination by a trained gynecologist at the time of recruitment in order to ascertain whether they had palpable tumors in pelvis, including myomas.

The present case and control subjects were all Japanese women who lived in Hokkaido, the island in the northern part of Japan. We used a self-answered questionnaire which consisted of demographic, reproductive and familial factors. With respect to the family history of myomas, we asked subjects if they had first-degree relatives of myomas; mother, sisters or daughters. In fact, subjects had neither daughters with myomas nor three and over first-degree relatives of myomas. If a woman did not completely answer to the questions, we immediately needed a personal or telephone interview for making up the incompleteness of data. Chi-squared test and unpaired Student’s t test were employed for statistical evaluation. The odds ratios (ORs), including 95% confidence intervals (CIs), and the linear trends were performed using the Mantel-Haenszel procedure and Mantel’s extension method^10^^,^^11^^)^.

## RESULTS

As seen in Table 1, the demographic factors of Japanese women with myomas were not different from those of healthy controls. However, myoma cases were more nulliparous than controls (23.6% versus 8.0%, respectively, Chi-squared test *p* < 0.01). Further, the percentage of positive family history of myomas in first-degree relatives among myoma patients is higher than that among controls (27.8% versus 17.0%, respectively, Chi-squared test *p* < 0.01), especially in two sisters (4.9% versus 0.7%, respectively, Chi-squared test *p* < 0.01).

**Table 1.** Demographic, reproductive and familial factors of 144 women undergoing hysterectomy or myomectomy for uterine myomas and 288 age-matched healthy controls.

	Cases			Controls		
Age (years)	44.66	±	4.92	44.63	±	5.48
Body mass index (kg/m^2^)	23.26	±	2.91	23.21	±	3.31
Education (years)	12.45	±	1.33	12.48	±	1.48
Oral contraceptives users (%)	5.0			3.0		
Smokers (%)	32.6			33.0		
Age at menarche (years)	13.12	±	1.27	13.06	±	1.38
Nulliparous (%)	23.6*			8.0		
Positive family history in first-degree						
relatives of myomas; mother or sisters (%)	27.8*			17.0		
One relative affected						
mother (%)	11.8			7.6		
sister (%)	9.0			7.3		
Two relatives affected						
mother and sister (%)	2.1			1.4		
two sisters (%)	4.9*			0.7		

*p < 0.01 compared with controls by using Chi-squared test.

Table 2. showed the ORs of parity. The risk of myomas was significantly reduced with the number of births. (Mantel’s extension method *p* < 0.01).

**Table 2.** Odds ratios of parity in 144 myoma cases and 288 controls.

	Cases	Controls	OR
	(n=144)	(n=288)	(95% CI)
Parity			
0	34	23	1.00
1	28	38	0.50 (0.24-1.02)
2	64	157	0.28 (0.15-0.50)
≧ 3	18	70	0.17 (0.08-0.36)
trend*			27.76**

* Chi-square value

** *p* < 0.01

Further, the ORs of parity according to the status of family history in mother or sisters of myomas are presented in Table 3. The risk of myomas decreased with the number of births, regardless of the status of familiality of myomas (Mantel’s extension method both *p* < 0.01).

**Table 3.** Odds ratios of parity in 144 myoma cases and 288 controls according to the positive or negative family history in first-degree relatives of myomas.

	Positive family history of myomas			Negative family history of myomas		
	
	Cases	Controls	OR	Cases	Controls	OR
	(n=40)	(n=49)	(95% CI)	(n=104)	(n=239)	(95% CI)
Parity						
0	8	2	1.00	26	21	1.00
1	6	6	0.25 (0.04-1.70)	22	32	0.56 (0.25-1.22)
2	22	28	0.20 (0.04-1.02)	42	129	0.26 (0.13-0.52)
≧ 3	4	13	0.08 (0.01-0.52)	14	57	0.20 (0.09-0.45)
trend*			7.62**			21.44**

* Chi-square value

** *p* < 0.01

In the subset women aged 45-54 years, the percentage of positive family history of myomas in first-degree relatives among myoma patients was higher than that among controls (31.5% versus 15.2%, respectively, Chi-squared test *p* < 0.01). However, there was no difference in this incidence between cases and controls in the subset women aged 30-45 years. In contrast, myoma patients were more nulliparous than controls in both younger and older subset groups (Chi-squared test *p* < 0.001 and *p* < 0.01, respectively) (Table 4).

**Table 4.** Distribution of the positive family history in first-degree relatives of myomas and nulliparous of 144 myoma cases and 288 controls according to age.

	Positive family history of myomas		Nulliparous	
	
	Cases n (%)	Controls n (%)	Cases n (%)	Controls n (%)
Age (years)				
< 45	11 (21.2)	24 (19.5)	18 (34.6)**	12 (9.8)
≧ 45	29 (31.5)*	25 (15.2)	16 (17.4)*	11 (6.7)

* *p* < 0.01 compared with controls by using Chi-squared test.

** *p* < 0.001 compared with controls by using Chi-squared test.

We categorized subjects into 4 groups by the status of the number of births (< 2 or ≧ 2) and the status of familiality of myomas (positive or negative). The results showed that OR was the greatest among women who had both fewer births and the positive family history in first-degree relatives of myomas as compared with those who had both more births and the negative familiality of myomas (OR = 5.8, 95% CI = 2.3-14.6) (Table 5). Furthermore, the tendency to increase OR was showed in nulliparous women with the familial aggregation of myomas (OR = 11.2, 95% CI = 2.3-53.8)

**Table 5.** Odds ratios by the status of parity and family history in first-degree relatives of myomas in 144 myoma cases and 288 controls (95% CI).

	Status of family history	

	Positive	Negative
Parity		
< 2	5.81 (2.32-14.57)	3.01 (1.84-4.92)
≧ 2	2.11 (1.19- 3.74)	1.00

## DISCUSSION

This study is concerned with the epidemiology of uterine leiomyomas in gynecology. To our knowledge, this is the first epidemiologic report with respect to the familial aggregation of uterine myomas in Japan.

In the present study, Japanese women undergoing surgical treatments for uterine myomas were enrolled as cases. Leiomyomas are benign tumors and often remain clinically silent throughout the reproductive life. Therefore, in an epidemiologic study of this disease, it seems to be important that myoma cases have the development of myomas which result in the indication of surgical treatments with a major public health problem. We assumed that women as cases who required hysterectomy or myomectomy for myomas almost always had myoma development.

In addition, appropriate controls appear to be crucial to the study. A case-control study for uterine myomas has a major bias toward the risk of the disease with respect to the control subjects. The reason for this is these benigh tumors are frequently asymptomatic through the reproductive life, therefore women with myomas unless they are checked by a doctor are likely to be recruited as controls. Hospital control subjects who are admitted for non-gynecological diseases or population-based controls may not be examined by a gynecologist for the presence/absence of myomas. This is the reason they may not have the complaint of menstruation such as menorrhagia and pelvic pain/pressure. Consequently, the controls may have asymptomatic myomas. On the other hand, hospital-based controls who had other gynecological diseases may have a bias toward the low fertility of myomas. If a woman is admitted for ovarian cancer, endometrial cancer or breast cancer, she may have fewer children in her reproductive life. It is best that the control subjects have not uterine myomas and furthermore, have not other women’s diseases which may be causative of a bias toward the fertility decline. Thus, we considered that healthy women who took part in a screening program for cervical cancer and breast cancer, with a past medical history, and had a pelvic examination by a trained gynecologist were eligible for recruitment as controls. Even if the healthy women had small myomas which were detected by ultrasound but not by bimanual pelvic examination, the myomas might be usually undeveloped ones. Therefore, it was assumed that a clinical or public health issue was rarely raised by these small tumors.

The myoma case and healthy control groups were similar demographically. However, Japanese women with uterine myomas tended to have mother or sisters reporting myomas. The findings are consistent with the previous studies that women with myomas have the familial aggregation of myomas^6^^,^^7^^)^. Race is unlikely to be associated with the familiality of myomas.

Although surgical intervention for these tumors is indicated through the reproductive years, the higher incidence of symptomatic myomas is observed in women ages 45-54^2^^)^. When the two age groups (30-44 years and 45-54 years) of myoma patients was considered, the positive family history of myomas in first-degree relatives was observed among middle-aged women with myomas. Younger adults myoma patients, on the other hand, had not significantly the familial aggregation of the disease, although the mechanism responsible for this remained unsettled. Our results suggest that fewer births are prone to be a risk for myomas consistently during the reproductive life. In contrast, Japanese women seem to have the familial predisposition to myomas in middle-age.

It is now recognized that there is an inverse relationship between the number of births and the chance of myoma development^8^^,^^9^^)^. In this study, the risk of uterine myomas increased with decreasing the parity among the subset groups of positive or negative family history of myomas in mother or sisters. These suggest that having fewer children may be a risk of uterine myomas in spite of the status of familiality of myomas. However, when data are analyzed according to the status of parity and first-degree familiality of myomas the risk of uterine myomas is the greatest among Japanese women with fewer births and the positive family history of myomas.

Genetic factors are likely to influence myoma formation in recent genetic studies, suggesting high mobility group protein genes may be crucial factors for the pathogenesis of leiomyomas^4^^,^^5^^)^. This is compatible with the finding of the familial predisposition to uterine myomas in this epidemiological study. Heritability in myomas may be of significance for the contributions to the etiology of the disease.

The present study failed to show the number of sisters in subjects. If patients with myomas had more sisters than controls, the study might result in the large number of case subjects with the familial aggregation of myomas. Further, we failed to know the ages of mother and sisters in subjects. Since the majority of uterine myomas are clinically apparent in their forties it may be necessary to take this characteristic into consideration for the familial risk in the study. If patients with myomas had more mother or sisters who ended their life in their younger days than controls, the research might result in the small number of case subjects with the familial predisposition to myoms. Therefore, further epidemiological studies, carefully designed, in the familial predisposition to myomas are needed for the future development of genetic aspects of uterine myomas.

In conclusion, Japanese middle-aged women with uterine myomas had the familial predisposition to myomas. More attention to myoma development may be of importance among nulliparous women who have the familial aggregation of uterine myomas.
